# Is microcephaly a so-far unrecognized feature of XYY syndrome?^☆^

**DOI:** 10.1016/j.mgene.2013.10.013

**Published:** 2014-01-31

**Authors:** Sylvie Nguyen-Minh, Christoph Bührer, Christoph Hübner, Angela M. Kaindl

**Affiliations:** a Department of Pediatric Neurology, Charité — Universitätsmedizin Berlin, Campus Virchow-Klinikum, Augustenburger Platz 1, 13353 Berlin, Germany; b Department of Neonatology, Charité — Universitätsmedizin Berlin, Augustenburger Platz 1, 13353 Berlin, Germany; c SPZ Pediatric Neurology, Charité — Universitätsmedizin Berlin, Campus Virchow-Klinikum, Augustenburger Platz 1, 13353 Berlin, Germany; d Institute of Neuroanatomy and Cell Biology, Charité — Universitätsmedizin Berlin, Campus Mitte, Charitéplatz 1, 10115 Berlin, Germany

**Keywords:** 47,XYY syndrome, Microcephaly, Mental retardation, Delayed speech development, Hyperactivity, Impulsiveness, array CGH, array comparative genomic hybridization, HAWIK-IV, Hamburg-Wechsler Intelligence test for Children, NCBI, National Center for Biotechnology Information, OFC, occipitofrontal head circumference, OMIM, Online Mendilian Inheritance in Man

## Abstract

• 47,XYY syndrome is a frequent sex chromosome aneuploidy.
• Overview of characteristic symptoms of 47,XXY
• First report of 47,XYY and microcephaly in a preterm child
• Brief differential diagnosis of microcephaly

To the Editor:

The 47,XYY syndrome is a frequent sex chromosome aneuploidy in males occurring in approximately 1/1000 male newborns (Ross et al., 2012). Patients characteristically exhibit a weight, height and head circumference above average starting at birth (Geerts et al., 2003), which has been associated with an overexpression of growth-related genes (Geerts et al., 2003, Korenberg et al., 1994). Attention has been raised to the presentation of delayed speech development and language based learning disabilities, increased frequency of attention deficit problems as well as hyperactivity and impulsiveness (Lalatta et al., 2012, Evans et al., 1986).

Here, we report a patient with a prenatally diagnosed 47,XYY karyotype who presented to our clinic at the age of 7 years with a secondary, early infantile microcephaly (Fig. 1). The index patient is the first child of healthy, unrelated parents. Amniocentesis was performed prenatally due to an increased nuchal fold, and chromosome analysis revealed a 47,XYY karyotype. A cesarean section was performed at 34 weeks of gestation due to preeclampsia with preterm labor and fetal heart rate abnormalities. At birth, the infant had a weight of 1930 g (40th centile), a length of 43 cm (40th centile) and a head circumference of 31 cm (50th centile). Birth was complicated by severe postnatal respiratory distress syndrome (Apgar 3/2, umbilical artery pH 7.32), requiring surfactant administration and mechanical ventilation for 9 days. Pre- and postnatal laboratory findings including infection parameters, microbiologic testing and newborn screening for metabolic and endocrine diseases were unremarkable. The patient did not show clinical signs of hypoxic-ischemic encephalopathy in the newborn period with continually normal neurological examination. Furthermore, normal brain morphology was confirmed by repeated cranial ultrasound and the background activity was normal on electroencephalography with no signs of epileptic discharges. Cardiologic morphology and function and ophthalmologic investigations at birth and at follow-up appointments were normal.

Microcephaly was noted at approximately 5 months of age (occipitofrontal head circumference (OFC) 40.5 cm, < 3rd centile). The motor milestones were normal, but a delay in speech development was noted. At the age of 7 years, the patient was presented in our clinic due to poor school performance and behavioral problems including, temper tantrums and aggressive behavior, refusal to perform tasks and an attention deficit that had become apparent shortly after school enrollment. A salient shyness with insecure behavior in a group setting and a negative self-perception were diagnosed in a psychological assessment. In the familial environment, the patient likewise exhibited outbursts of rage and refusal in reaction to frustrating events and an enormous fear of failure. Cognitive ability testing revealed intellectual impairment with learning disability (Hamburg-Wechsler Intelligence test for Children (HAWIK-IV): overall cognitive ability 79, verbal comprehension index 88, perceptual reasoning index 90, processing speed index 74 and working memory index 80) and marked difficulties in auditory discrimination and short-time memory. Clinical examination at age 7 was unremarkable except for a microcephaly (OFC 50 cm, < 3rd centile), a mild gait ataxia without further coordination deficit and a non-palpable left testis (body weight 15th centile, body length 40th centile). We confirmed a 47,XYY karyotype in a repeated chromosomal analysis and ruled out further microdeletions or -duplications through array CGH analysis (Microarray Kit 244A, Agilent Technologies, Santa Clara, USA) from the index patient.

To our knowledge, we here report the first patient with a 47,XYY karyotype and a secondary, early infantile microcephaly rather than a normal head circumference or macrocephaly reported previously in this context (Nicolson et al., 1998, Fryns et al., 1995, Ratcliffe et al., 1994, Ottesen et al., 2010, Ratcliffe et al., 1979, Evans et al., 1986). Similarly, weight and height of our patient are in the lower normal range, while an increase in both body measurements has been reported for patients with XYY syndrome (literature search in the NCBI and OMIM databases with the search terms ‘47,XYY’ or ‘sex chromosome abnormalities’ in combination with ‘head circumference’, ‘body measurements’, ‘centiles’, ‘growth’, ‘microcephaly’, ‘macrocephaly’ as well as ‘effect of chromosome abnormalities on growth’). Hypoxic-ischemic encephalopathy and exogenous causes of microcephaly such as *in utero* exposure to alcohol, malnutrition or perinatal infection were excluded. Genetic testing was repeated postnatally and complemented by an array CGH to exclude additional numerical chromosome aberrations or microdeletion or -duplication syndromes that could cause the microcephaly in our index patient. Among the numerous genetic syndromes associated with microcephaly, we specifically aimed at excluding a trisomy of chromosome 21, since this has been linked to both the 47,XYY karyotype (Fryns et al., 1995) and microcephaly (Korenberg et al., 1994). Underlying cardiologic dysfunction was excluded as a cause of dystrophy. Moreover, no severe asphyxia occurred as a cause of the patient´s secondary microcephaly. The pattern of behavioral problems reported in our patient is in line with the previously reported increased risk of impulsivity, poor adaptation to social situations and attention or hyperactivity disorder (Ross et al., 2012). The learning disability of our patient is concurrent with a previously reported prevalence of up to 80% in 47,XYY syndrome (Geerts et al., 2003). Our index patient also showed a marked deficit in auditory discrimination, which might be a contributing factor to the high prevalence of language development delay in 47,XYY syndrome (Geerts et al., 2003).

In conclusion, our report shows that 47,XYY karyotype can be associated with a microcephaly and broadens the spectrum of clinical features. The behavioral problems described in our patient are in line with previous reports. Accordingly, clinicians may also consider an underlying 47,XYY syndrome in a patient with the “typical pattern” of behavioral problems, a microcephaly and average or low body measurements. Further follow-up of children with 47,XYY karyotype born preterm will have to be undertaken to provide a reference of expectable body measurements.

## Figures and Tables

**Fig. 1:**
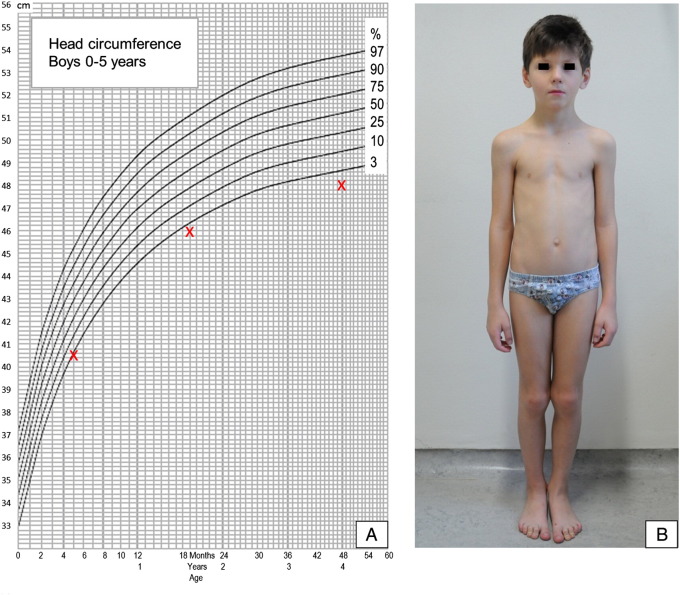
Clinical phenotype of the patient. (A) Head circumferences of the index patient (head circumference chart according to longitudinal studies in Zürich, Switzerland from 1974 to 2009, Pediatrica 2011, Vol. 22, No. 1). (B) Picture of the index patient at age 7 illustrating microcephaly.
